# Therapeutic efficacy of tumor-targeting *Salmonella typhimurium* A1-R on human colorectal cancer liver metastasis in orthotopic nude-mouse models

**DOI:** 10.18632/oncotarget.5187

**Published:** 2015-09-07

**Authors:** Takashi Murakami, Yukihiko Hiroshima, Ming Zhao, Yong Zhang, Takashi Chishima, Kuniya Tanaka, Michael Bouvet, Itaru Endo, Robert M. Hoffman

**Affiliations:** ^1^ AntiCancer, Inc., San Diego, California, USA; ^2^ Department of Surgery, University of California San Diego, California, USA; ^3^ Department of Gastroenterological Surgery, Graduate School of Medicine, Yokohama City University, Yokohama, Japan

**Keywords:** nude mice, orthotopic, liver metastasis, red fluorescent protein, *Salmonella typhimurium* A1-R

## Abstract

Liver metastasis is the most frequent cause of death from colon and other cancers. Generally, liver metastasis is recalcitrant to treatment. The aim of this study is to determine the efficacy of tumor-targeting *Salmonella typhimurium* A1-R on liver metastasis in orthotopic mouse models. HT-29 human colon cancer cells expressing red fluorescent protein (RFP) were used in the present study. *S. typhimurium* A1-R infected HT-29 cells in a time-dependent manner, inhibiting cancer-cell proliferation *in vitro. S. typhimurium* A1-R promoted tumor necrosis and inhibited tumor growth in a subcutaneous tumor mouse model of HT-29-RFP. In orthotopic mouse models, *S. typhimurium* A1-R targeted liver metastases and significantly reduced their growth. The results of this study demonstrate the future clinical potential of *S. typhimurium* A1-R targeting of liver metastasis.

## INTRODUCTION

Anecdotal records go back hundreds of years describing patients having their cancer go into remission after a bacterial infection [1]. In 1867, the German physician Busch reported that a cancer patient went into remission after contracting erysipelas, now known as *Streptococcus pyogenes* [2]. Bruns treated a cancer patient in 1888 with *S. pyogenes* and the tumor regressed [1]. Koch, Pasteur and von Behring recorded that cancer patients infected with *S. pyogenes* had tumor regression [1].

In the 1890s, William B. Coley of New York Cancer Hospital, which became Sloan-Kettering Memorial Cancer Center, treated cancer patients with *S. pyogenes*. Coley had excellent results infecting cancer patients with *S. pyogenes.* Hoption Cann et al. [3] compared Coley's bacterial treatment to current chemotherapy and found the 10-year survival rates of Coley's patients were comparable [4] to current conventional therapies [4].

Malmgren and Flanigan [5] demonstrated that anaerobic bacteria could survive and replicate in necrotic tumor tissue which had low oxygen content. Several other early approaches aimed at utilizing bacteria for cancer therapy in animal models were described [6–16].

The obligate anaerobes *Bifidobacterium* [17] and *Clostridium* [18], which replicate only in necrotic areas of tumors, have been tested for cancer therapy in mouse models. *Bifidobacterium longum* selectively localized in mammary tumors after systemic administration [17]. Spores of *Clostridium novyi*, without its lethal toxin (*C. novyi* no toxin [NT]). germinated within necrotic areas of tumors in mice and, in combination with chemotherapy, resulted in hemorrhagic necrosis and tumors regression [18]. Recently, *C. novyi*-NT was used in a patient with leiomyosarcoma and caused one metastatic lesion to regress [19].

*Salmonella typhimurium* (*S. typhimurium*) is a facultative anaerobe which, in contrast to obligate anaerobes, can grow in the viable regions as well as necrotic regions of tumors [20]. *S. typhimurium* with a lipid A—mutation (msbB) deletion along with purine auxotrophic mutations (purI) had antitumor efficacy in mice [21]. *S. typhimurium* (VNP20009), with msbB and purI mutations, was relatively safely administered to patients in a Phase I clinical trial on patients with metastatic melanoma and renal carcinoma. Overattenuation perhaps limited efficacy [22].

Liver metastases is the most frequent cause of death of patients with colorectal cancer [23]. Hepatectomy is the most effective treatment for liver metastasis from colorectal cancer, but the recurrence rate is over 50% after resection [24]. In addition, efficacy of chemotherapeutic agents are marginal [25]. Therefore, development of effective treatment for liver metastasis is urgently needed.

The tumor-targeting *S. typhimurium* A1-R strain developed by our laboratory has high tumor colonization and antitumor efficacy. *S. typhimurium* A1-R is auxotrophic for leu-arg, which prevents it from mounting a continuous infection in normal tissues. *S. typhimurium* A1-R was able to inhibit or eradicate primary and metastatic tumors as monotherapy in nude mouse models of prostate [26, 27], breast [28–30], lung [31, 32], pancreatic [33–37], ovarian [38, 39], stomach [40] and cervical cancer [41], as well as sarcoma [42–45] and glioma [46, 47], all of which are highly aggressive tumor models.

The present report demonstrates efficacy of *S. typhimurium* A1-R on liver metastasis of colon cancer in orthotopic mouse models.

## RESULTS AND DISCUSSION

### *S. typhimurium* A1-R targeted HT-29 colon cancer cells *in vitro*

**Figure 1:**
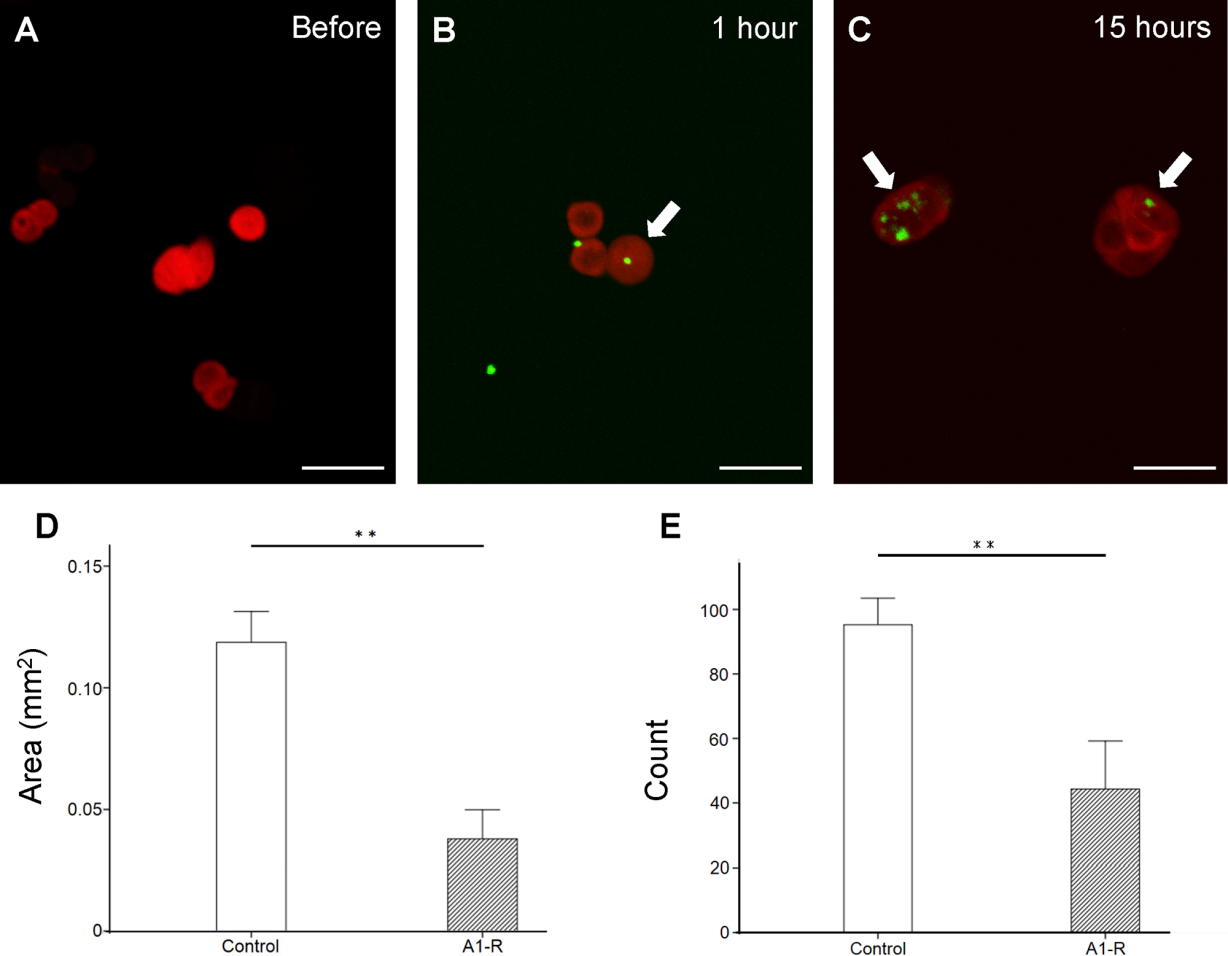
*In vitro* efficacy of *S. typhimurium* A1-R-GFP on HT-29-RFP colon cancer cells **A**–**C.** Confocal imaging of HT-29-RFP cells with *S. typhimurium* A1-R-GFP over time with the FV1000 confocal microscope. *S. typhimurium* A1-R infection of HT-29-RFP cells at one hour after administration (B) At 15 hour after administration, more bacterial cells were visualized in cancer cells (C). Arrows show infecting *S. typhimurium* A1-R expressing GFP. *S. typhimurium* A1-R inhibited cell proliferation both in colony area **D.** and number **E.** **P < 0.01. Error bars: ± 1 SE. Scale bars: 20 μm (BF, bright-field). The cells in Figures 1A, B and C were chosen as before and after examples of infection with *S. typhimurium* A1-R-GFP, not to indicate efficacy, which occurs at later times.

*S. typhimurium* A1-R-GFP infection of HT-29 cells expressing red fluorescent protein (HT-29-RFP) was observed beginning 1 hour after addition of bacteria to the cultures (Figure 1A–1B). At 15 hours after addition, many *S. typhimurium* A1-R-GFP cells were observed inside the HT-29 cancer cells (Figure 1C).

### *S. typhimurium* A1-R inhibited HT-29 cancer cell proliferation *in vitro*

HT-29-RFP cancer cell colonies were significantly reduced by *S. typhimurium* A1-R-GFP both in area (Figure 1D) and number (Figure 1E) (*P* < 0.01 for both).

### Efficacy of *S. typhimurium* A1-R on subcutaneous HT-29 tumor growth

**Figure 2:**
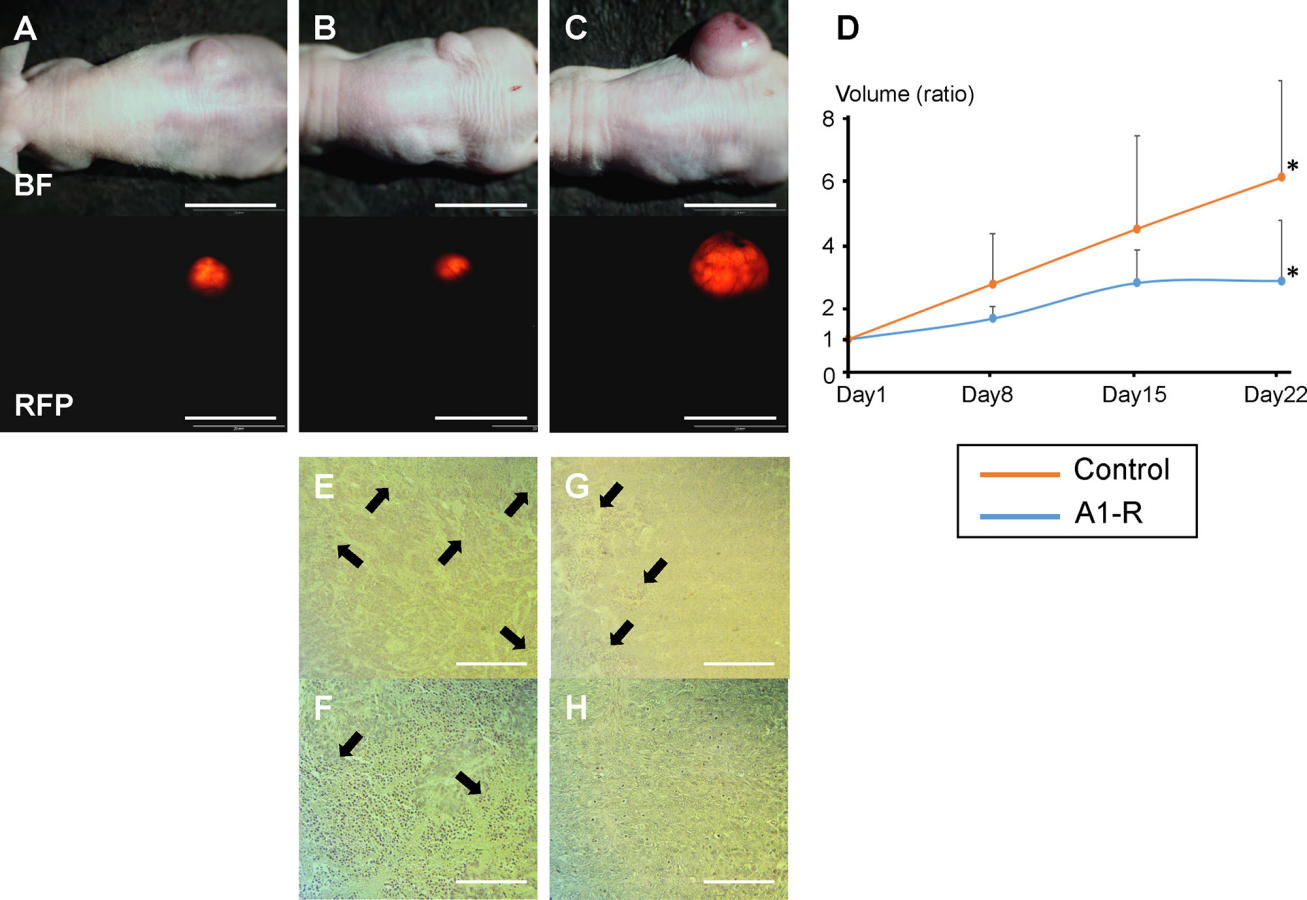
Efficacy of *S. typhimurium* A1-R on HT-29-RFP subcutaneous tumor growth Subcutaneous tumor models were established by injection of HT-29-RFP cells in the flanks of nude mice. **A**–**C.** Upper panels show bright-field images of tumor growth and lower show RFP images of tumor growth obtained with the OV-100 Small Animal Imaging System. An HT-29-RFP subcutaneous tumor in the right flank before treatment (day 1) (A), and after treatment with S. *typhimurium* A1-R at day 22 (B), HT-29-RFP tumor in the untreated control group at day-22 (C). **D.** *S. typhimurium* A1-R administration significantly decreased tumor volume at day-22 compared to no treatment. **E**–**H.** Representative histological images of excised tumors. *S. typhimurium* A1-R treated tumors had scattered necrosis surrounding viable cancer (E), (F). In contrast, untreated tumors had less necrosis (G), (H). (F) and (H) are high-magnification images of (E) and (G), respectively. *P < 0.05. Error bars: ± 1 SE. Arrows show necrotic areas. Scale bars: 20 mm (A) – (C), 500 μm (E) and (G), 200 μm (F) and (H).

Subcutaneous tumor growth was significantly inhibited after the 3^rd^ i.v. administration of *S. typhimurium* A1-R. The tumor volume ratio at day 22 compared to day 1 in the control group was 6.17 ± 1.16 and 2.68 ± 0.73 in the *S. typhimurium* A1-R treatment group, *P* < 0.05 (Figure 2A–2D). Resected specimens showed *S. typhimurium* A1-R induced more extensive necrosis in the tumors compared to those without treatment (Figure 2E–2H).

### Efficacy of *S. typhimurium* A1-R on orthotopic liver metastasis mouse models

**Figure 3:**
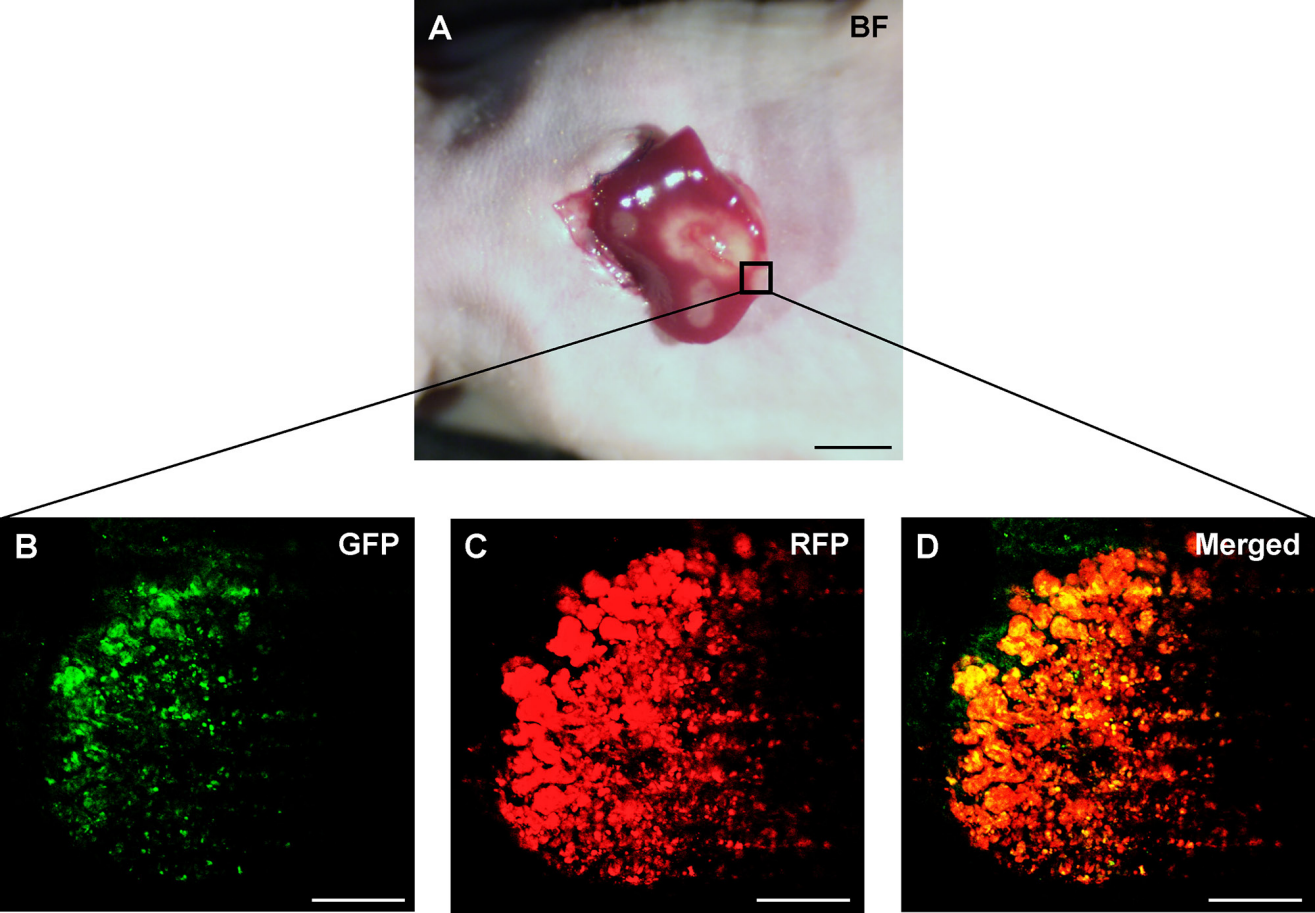
Intravital imaging of tumor-targeting *S. typhimurium* A1-R in HT-29 liver metastasis *S. typhimurium* A1-R-GFP was visualized in the HT-29-RFP liver metastases at day 11 (three days after the second administration of *S. typhimurium* A1-R-GFP). **A.** Liver metastases were visualized with the OV100. **B–D.** Confocal imaging with the FV1000 demonstrated *S. typhimurium* A1-R-GFP targeting the HT-29-RFP liver metastasis. Scale bars: 5 mm (A), and 50 μm (B)–(D).

**Figure 4:**
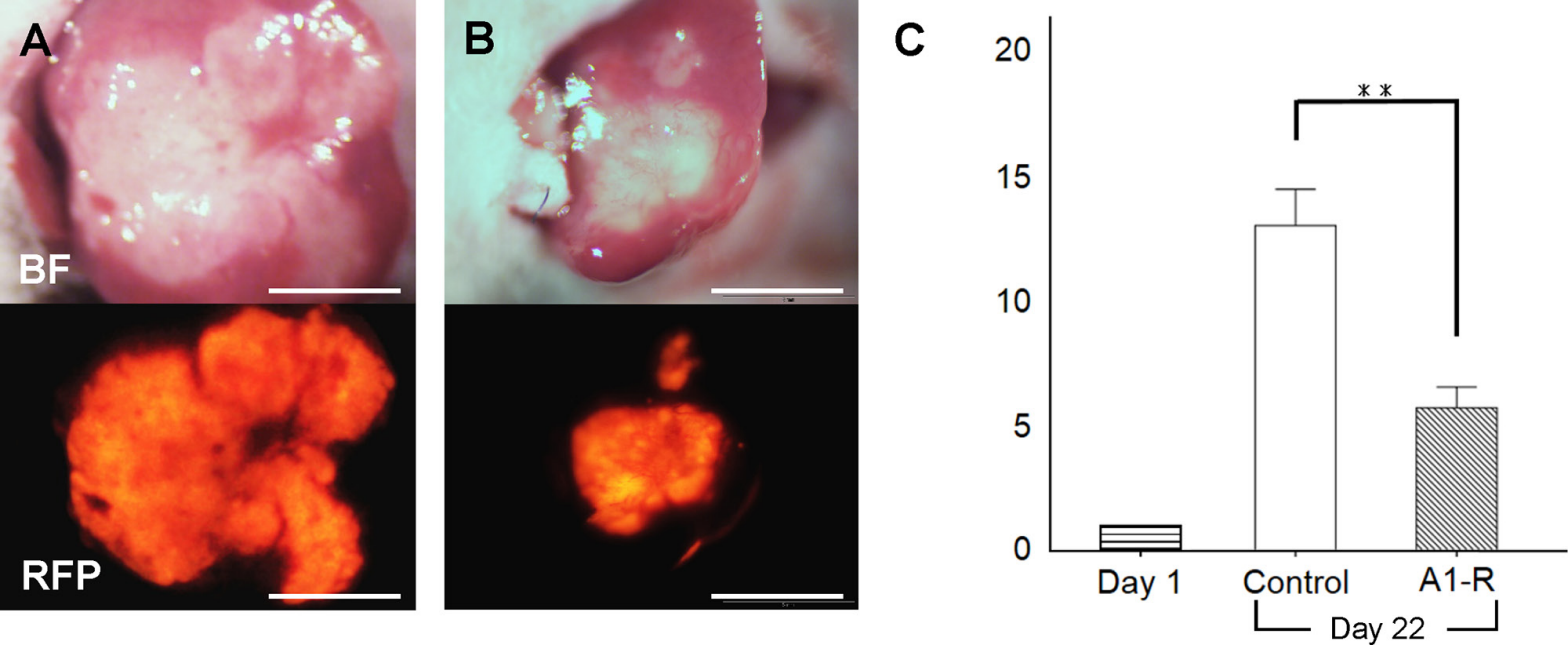
Efficacy of *S. typhimurium* A1-R on HT-29-RFP liver metastasis **A, B.** Upper panels are bright-field and lower panels are RFP images. Images of liver metastasis at day-22. No treatment (control group; A) and treated with *S. typhimurium* A1-R (*S. typhimurium* A1-R group; B). **C.** Bar graphs demonstrates the ratio of tumor fluorescent area at day 22 to day 1. Metastasis growth in the A1-R group was significantly inhibited compared to the untreated control group. **P < 0.01. Scale bars: 5 mm.

*S. typhimurium* A1-R-GFP targeted the liver metastasis 3 days after the 2^nd^ i.v. administration of *S. typhimurium* A1-R (Figure 3). *S. typhimurium* A1-R treatment significantly suppressed metastatic progression compared to the control group at day 22. The metastasis fluorescent area ratio on day 22 compared to day 1 was 5.69 ± 0.83 in the *S. typhimurium* A1-R treatment group and 12.96 ± 1.49, in the untreated control group (*P* < 0.01; Figure 4).

The present study demonstrated that tumor-targeting *S. typhimurium* A1-R has significant efficacy on liver metastasis in orthotopic mouse models, suggesting clinical activity for patients with colorectal liver metastasis. The present study suggests *S. typhimurium* A1-R may also be useful in the neo-adjuvant setting to reduce the liver metastasis that would make previously inoperable liver metastasis resectable.

Spleen injection selects colon cancer cells which can grow in the liver. However, this leads to multiple metastatic sites, which were harvested to directly implant a single metastases on the liver of additional mice, which was the desired model. Future experiments will treat models with multiple liver metastases with *S. typhimurium* A1-R.

This is the first study to use *S. typhimurium* A1-R to treat colon cancer liver metastasis, which occurs at high frequency and is usually the cause of lethality of this disease. The significant efficacy of *S. typhimurium* A1-R on the liver metastasis was comparable to the efficacy of *S. typhimurium* A1-R on other tumor types [26–47].

We have previously demonstrated efficacy of *S. typhimurium* A1-R in combination with anti-angiogenesis therapy in pancreatic cancer mouse models [37]. Future experiments will also test this combination in colon-cancer liver-metastasis models.

*S. typhimurium* A1-R in combination with chemotherapy was also active against stomach and pancreatic cancer models [35, 40]. Such strategies will also be tested on colon-cancer liver-metastasis in future experiments.

The present study used GFP and RFP to image *S. typhimurium* A1-R and the cancer cells, respectively, *in vitro* and *in vivo*. Genetic reporters have an important advantage over injectable probes in that the label is permanent with genetic reporters. This is important for long-term monitoring of bacterial targeting as well as tumor growth, including metastasis as well as recurrence [48].

Bacteria have numerous advantages over other biological therapy in that bacteria actively invade tumors, even when vascularity is poor and have a large genome to manipulate for effective and selective tumor targeting [2].

Previously developed concepts and strategies of highly selective tumor targeting [49–53] can take advantage of bacterial targeting of tumors.

Tissue-selective therapy focuses on unique properties of normal tissues and how therapy can target a property of a tissue that kills the cancer tissues that arise from the normal tissue without affecting other tissues [49, 54]. *S. typhimurium* A1-R is an example of tissue-selective therapy.

De-differentiation of a tumor leading to resistance to chemotherapy is a limitation of tissue-selective therapy, since the targeted protein or pathway may no longer be expressed in the de-differentiated tumor [54]. However, *S. typhimurium* A1-R does not seem to depend on such targets and should be active against de-differentiated tumors. Specific caspase inhibitors may protect normal cells which may not be present in drug-resistant cancer cells and those could provide further protection to normal cells during *S. typhimurium* A1-R therapy [49, 51]. *S. typhimurium* A1-R may also be effectively combined with teratogens which could selectively effect cancer cells that are de-differentiated [50]. Since *S. typhimurium* A1-R can decoy quiescent cancer cells to begin to cycle, *S. typhimurium* A1-R could be effectively combined with agents which selectively target proliferating cancer cells [52]. In this regard, normal cells could be protected by agents which induce wild type p53 [53].

## MATERIALS AND METHODS

### Cell line

The human colon cancer cell line HT-29 [55, 56] was maintained in DMEM (Irvine Scientific, Irvine, CA) supplemented with heat-inactivated 10% fetal bovine serum (FBS) (Gemini Biologic Products, Calabasas, CA), 2 mM glutamine, 100 units/ml penicillin, 100 μg/ml streptomycin, and 0.25 μg/ml amphotericin B (Life Technologies, Inc., Grand Island, NY). The cells were incubated at 37°C in 5% CO_2_. Expression of RFP indicated viability.

### Establishment of RFP-labeled HT29

The pDsRed-2 vector (Clontech Laboratories Inc., Palo Alto, CA) expressing RFP and neomycin resistance gene were used to transfect HT-29 to stably express RFP. For RFP gene transfection, 25% confluent HT-29 cells were incubated with a mixture of retroviral supernatants of PT67-RFP packaging cells and DMEM for 24 h. Fresh medium was replenished at this time, and cells were allowed to grow in the absence of retrovirus for 12 h. This procedure was repeated until high levels of RFP expression were achieved. Cells were then harvested with trypsin-EDTA and subcultured into selective medium that contained 200 μg/ml G418 (Invitrogen Corp., Carlsbad, CA). The level of G418 was increased to 2, 000 μg/ml stepwise. HT-29 clones expressing high levels of RFP were isolated and were amplified and transferred using conventional culture methods. High RFP-expression clones were subsequently isolated in the absence of G418 for 10 passages to select for stable expression of RFP [55, 57, 58].

### Mice

Athymic *nu/nu* nude mice (AntiCancer Inc., San Diego, CA), 4–6 weeks old, were used in this study. All mouse surgical procedures and imaging were performed with the animals anesthetized by subcutaneous injection of a ketamine mixture (0.02 ml solution of 20 mg/kg ketamine, 15.2 mg/kg xylazine, and 0.48 mg/kg acepromazine maleate). All animal studies were conducted in accordance with the principles and procedures outlined in the National Institutes of Health Guide for the Care and Use of Animals under Assurance Number A3873-1.

### Preparation of *S. typhimurium* A1-R

GFP-expressing *S. typhimurium* A1-R bacteria (AntiCancer Inc.,) were grown overnight on LB medium (Fisher Sci., Hanover Park, IL, USA) and then diluted 1:10 in LB medium. Bacteria were harvested at late-log phase, washed with PBS, and then diluted in PBS [28].

### Clonogenic assay

HT-29-RFP cells (2.0 × 10^2^) were seeded in 35 mm dishes. *S. typhimurium* A1-R-GFP (5 × 10^5^ CFU/ml) was added to the cancer cells, which were incubated at 37°C for 40 minutes. The cells were rinsed and cultured in medium containing gentamycin (10 μg/ml). After 9 days culture, the colonies were fixed with methanol and stained with crystal violet. The Image J program v1.49f (National Institutes of Health) was used to quantify the colonies.

### Confocal imaging of cancer cells infected by *S. typhimurium* A1-R

HT-29-RFP cells (4.0 × 10^4^) were seeded in 35 mm dishes. *S. typhimurium* A1-R-GFP (5 × 10^5^ CFU/ml) was added to the cancer cells, which were incubated at 37°C for 40 minutes. The cells were rinsed and cultured in medium containing gentamycin (10 μg/ml). The interaction between *S. typhimurium* A1-R expressing GFP and HT-29 cancer cells expressing RFP was imaged in real time by confocal microscopy (Fluoview FV1000, Olympus Corp., Tokyo, japan) [59] before, and 1 hour and 15 hours after administration of *S. typhimurium* A1-R.

### Efficacy of *S. typhimurium A1-R* on HT-29 subcutaneous tumors

HT-29-RFP cells were harvested by trypsynization and washed with serum-free medium. A cell suspension (2 × 10^6^ cells/100 μl in medium with 50% Matrigel) was injected subcutaneously in the right flank of nude mice. Two weeks after cell injection, established subcutaneous tumors were measured weekly and treated. Fourteen mice were randomized into a control group (*n* = 7) and an *S. typhimurium* A1-R treatment group (*n* = 7). The first treatment day was defined as day 1. Mice in the *S. typhimurium* A1-R group were treated with *S. typhimurium* A1-R (5 × 10^7^ CFU/body) 3 times at days 1, 8, and 15. All tumors in both groups were observed weekly with the OV100 Small Animal Imaging System (Olympus) [60] and harvested at day 22 for tissue evaluation. Each tumor was measured individually in each mouse. Tumor volume was calculated with the following formula: Tumor volume = 1/2 × Length × Width^2^. Treatment efficacy was presented as a ratio of the tumor volume at each time point compared to the tumor volume at the beginning of the treatment. Dosing was determined from efficacy in other tumor models of *S. typhimurium* A1-R [26–47].

### Initial establishment of liver metastases

**Figure 5:**
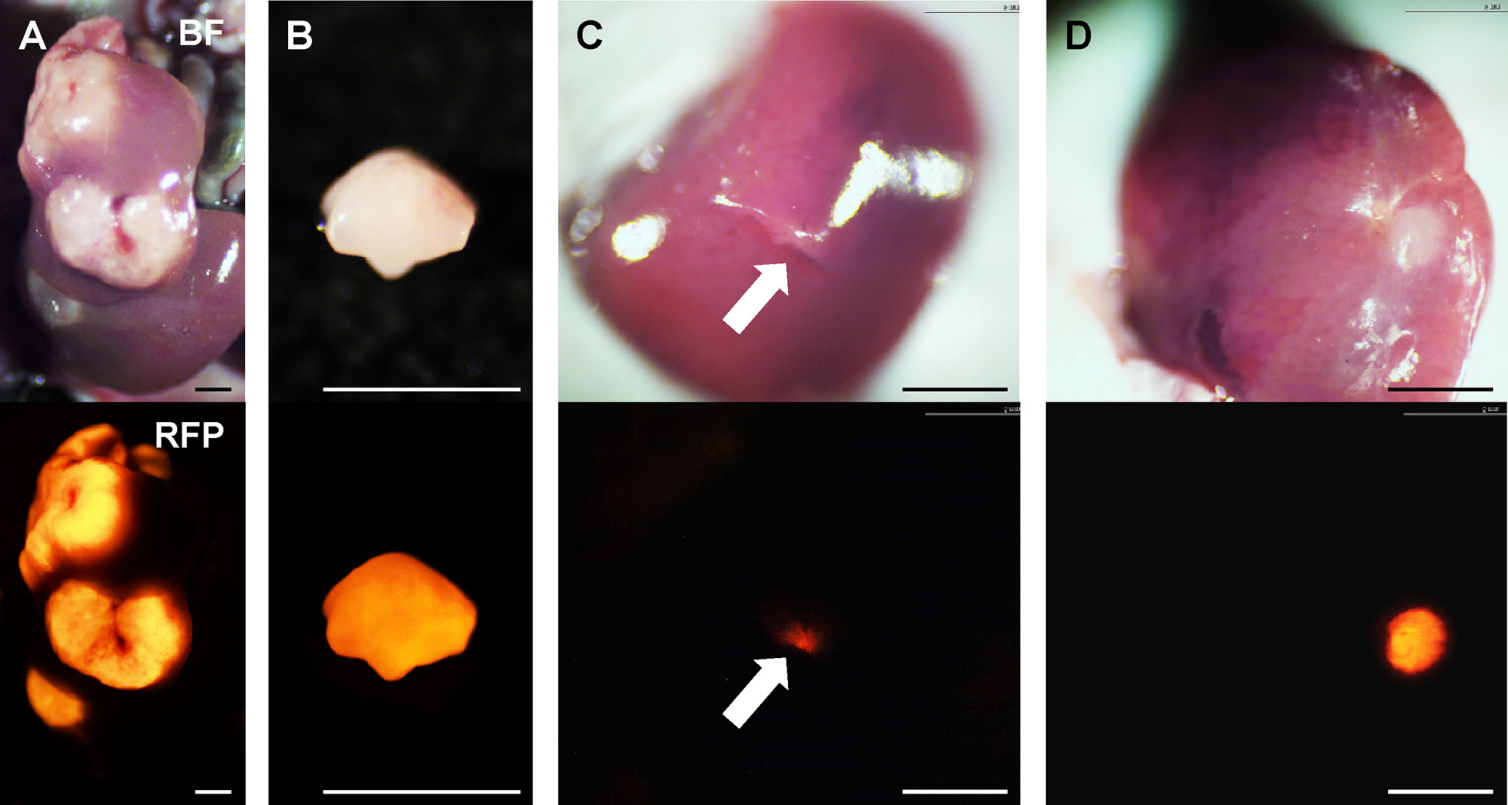
Establishment of orthotopic liver metastasis mouse models Upper panels show bright-field images and lower are RFP images. **A.** Multiple liver metastases were initially established after spleen injection of HT-29-RFP cells in the donor mouse. **B.** The metastasis were resected and cut into small fragments. **C.** Single fragments were then orthotopically implanted in the left lobe of the liver in the experimental mice through an incision (arrow). **D.** Four weeks after implantation, an orthotopic liver metastasis mouse model was established. Scale bars: 3 mm.

HT-29-RFP cells were harvested by trypsinization and washed twice with serum-free medium. HT-29-RFP cells (5 × 10^5^ in 50 μl serum-free medium with 50% Matrigel) were injected into the superior and inferior pole of the spleen in nude mice. Three weeks after injection, liver metastases were established (Figure 5A).

### Surgical orthotopic implantation of liver metastasis

Liver metastases, as described above, were resected and cut into block (8 mm^3^) (Figure 5B). A single tumor fragment was orthotopically implanted into the left lobe of the liver in other nude mice (Figure 5C). Four weeks later, liver metastasis were observed at the implanted site (Figure 5D).

### Efficacy of *S. typhimurium* A1-R on liver metastasis

Four weeks after orthotopic implantation of HT-29-RFP to the liver, 10 mice were randomized into 2 groups: untreated control group (weekly, 3 weeks, *n* = 5) and the *S. typhimurium* A1-R treatment group (*S. typhimurium* A1-R, 5 × 10^7^ CFU/body, iv, weekly, 3 weeks, *n* = 5). The left lobe of the liver with metastasis was exposed before (at day 1) and after treatment (at day 22) for observation with the OV100. The tumor fluorescence area was analyzed with ImageJ software. Treatment efficacy in each mouse was compared as a ratio of the tumor volume at each time point compared to the tumor volume at the beginning of treatment. Liver metastasis in the *S. typhimurium* A1-R treatment group was imaged with the FV1000 confocal microscope at day 11 to observe *S. typhimurium* A1-R-GFP targeting the RFP-expressing HT-29 liver metastasis.

### Histology of tissue

Fresh tumor samples were fixed in formalin (10%) and embedded in paraffin before sectioning and staining. Tissue sections (3 mm) were deparaffinized in xylene and rehydrated in an ethanol series. Hematoxylin and eosin (H & E) staining was performed according to standard protocols.

### Statistical analysis

SPSS statistics version 21.0 was used for all statistical analyses (IBM, New York City, NY, USA). Significant differences for continuous variables were determined using the Mann-Whitney U test. Bar graphs expressed average values and error bar showed SE. A probability value of *P* ≤ 0.05 was considered statistically significant.
